# Perceptions of and Satisfaction with Sexual and Reproductive Health Interventions in Pre-Adolescent and Adolescent Students in UE/EEA Countries: A Systematic Review

**DOI:** 10.3390/healthcare11070939

**Published:** 2023-03-24

**Authors:** Marco Montalti, Yari Longobucco, Chiara Celani, Laura Dallolio, Alice Masini

**Affiliations:** 1 Department of Biomedical and Neuromotor Science, University of Bologna, 40126 Bologna, Italy; 2 Department of Health Sciences, University of Florence, 50134 Florence, Italy

**Keywords:** sexual health, education, peer to peer, review, qualitative, public health

## Abstract

The objective of this review is to investigate perceptions of and satisfaction with sexual and reproductive health (SRH) interventions among pre-adolescents and adolescents of all genders and ethnicities in EU/EEA countries. This systematic review was conducted in accordance with PRISMA recommendations. A systematic literature search was conducted on MEDLINE (PubMed), Cochrane Central Register of Controlled Trials (Central), CINAHL (EBSCO), and PsycINFO (EBSCO) up to March 2022 to identify all published articles that included information on perceptions and levels of satisfaction with SRH interventions. The selected studies were independently and blindly evaluated for risk of bias. Finally, only five papers were included in our review, divided according to the implemented intervention design: conducted by research groups (health or non-health professionals) or structured entirely in a peer-to-peer approach. Both types of program designs analyzed in the included studies were well accepted by students, even though satisfaction assessment methods were not standardized. Peer-to-peer conducted SRH programs or interventions with practical components (e.g., exercises, discussion) were more appreciated. We also found higher levels of satisfaction among younger participants. Future SRH educational programs should consider an assessment of participants’ perceptions and satisfaction, possibly adopting standardized tools. Following a peer-to-peer structure and delivering programs early could lead to greater participant satisfaction.

## 1. Introduction

Sexual and Reproductive Health (SRH), according to the United Nations Population Fund (UNFPA), encompasses all issues related to the reproductive system: from physical to mental and social well-being. It implies that people can have a satisfying and safe sex life, the ability to reproduce, and the freedom to decide if, when, and how often to do so [1]. The European office of the World Health Organization (WHO/Europe) is constantly updated on the SRH concerns of the countries assisted to evaluate the situations and to find optimal ways to improve them. As SRH is a very personal subject, people may have trouble finding or asking for accurate information about it. For WHO/Europe, this may help explain why some issues are still not addressed openly and services are inadequate, fragmented, and unfriendly in some countries across the European region. Complications of pregnancy and childbirth, unsafe abortions, reproductive tract infections, sexual violence, and women dying from avoidable cancer are just a few of the problems in the area [2]. 

In order to have good SRH, people must have access to accurate information. Indeed, in recent decades, it has been increasingly recognized and proven that teaching the cognitive, emotional, social, and physical aspects of sexuality can have a positive impact on the SRH of children and young people. This is also why, in 2021, the European Commission published a report describing the current state of sexuality education aimed at adolescents and pre-adolescents within member states, finding that, as of November 2019, it was mandatory in 19 Member States for schools to offer some sort of sexuality education, while this remained optional in a further 8 Member States. In addition, in some countries (such as Cyprus, Italy, Romania, and Slovenia), SRH education programs focus largely on biological elements alone [3]. In a recent review of the effectiveness of SRH education programs, Corcoran et al. found that adolescents seek external sources of education, such as peers and the media, when the content provided by school educators or health professionals is deemed irrelevant or the education is perceived as biased [4,5,6]. This tendency to rely on the media as an SRH informational source has been increasing in recent decades [5,6]. It is with the aim of creating effective programs that the United Nations Educational, Scientific and Cultural Organization (UNESCO) published a technical report on sexuality education in 2018. Within the report, aspects of Comprehensive Sexual Education (CSE) are better outlined, as a “curriculum-based teaching and learning process on the cognitive, emotional, physical and social aspects of sexuality” [7]. CSE includes scientifically accurate information about human development, anatomy, and reproductive health, as well as information about contraception, childbirth, and sexually transmitted infections (STIs), including HIV. However, CSE also goes beyond information, helping young people to explore and nurture positive values regarding their sexual and reproductive health and rights [1]. 

The UNESCO report also includes a specific section on CSE programs’ evaluation, as monitoring the effectiveness in terms of knowledge gained and changes in the behaviors of the recipients is, indeed, of the utmost importance. However, within the report, it is emphasized how an evaluation of the perception of and satisfaction with a CSE program would also be of great importance, especially considering the previously mentioned findings by Corcoran et al. [7]. Despite the importance of evaluation stressed by international organizations, which is essential for continuous improvements in the adopted tools, there are not many examples in the international scientific literature that specifically focus on this phase of programs. In a 2020 review by Ivanova et al., the authors summarized the available scientific literature on the evaluation designs used for SRH education programs, focusing only on low- and middle-income countries. The analysis showed how evaluations are largely dominated by quantitative experimental designs and the use of public health outcomes [8]. However, in order to improve SRH education programs, it is important to evaluate not only the quality of program development, implementation, and impact but also to measure the perceptions and satisfaction of the users involved in the programs.

Qualitative studies often provide more detailed insight and capture shades that may be missed using quantitative surveys.

In some studies conducted in recent years, perceptions and satisfaction with this type of intervention have been found to be generally high: Kamke et al., in the United States, found that 92 percent of program participants would recommend the intervention to a friend and 98 percent would use what they learned in the future [9]; in another study, conducted in Canada by Meaney et al., general satisfaction with school-based sexual health education was found, particularly with regard to changes in knowledge and values, and a preference for exposure to earlier classes [10].

The objective of this review is to investigate perceptions of and satisfaction with sexual health interventions in school and community settings of pre-adolescents and adolescents of all genders, sexes, and ethnicities in EU/EEA countries.

## 2. Materials and Methods

### 2.1. Search Strategy

This systematic review was conducted in accordance with PRISMA recommendations and the criteria of the reporting of systematic review and meta-analysis guidelines [11]. The systematic review protocol was registered in the International Prospective Register of Systematic Reviews. The following PICO (Patients, Interventions, Comparators, and Outcomes) question was developed, addressing the primary search objective, through the following search terms: (P) pre-adolescents and adolescents at secondary school and high school of any gender, sex, and ethnicity; (I) sexual health interventions in school and community setting (any time and frequency), including both the prevention of unintended pregnancies and STIs; (C) both studies with or without control groups—the control groups could be involved in no activity based on sexual health intervention or different types of intervention; (O) perceptions and level of satisfaction with sexual health interventions for pre-adolescent and adolescent students. A systematic literature search of MEDLINE (PubMed), Cochrane Central Register of Controlled Trials (Central), CINAHL (EBSCO), and PsycINFO (EBSCO) up to March 2022 was conducted to identify all published articles, including information about the perceptions and levels of satisfaction with SRH interventions for pre-adolescent and adolescent students. We searched electronic databases, with a 10-year publication date limit, because we were interested in recent approaches. The following criteria were used to define our research: we included only Randomized Controlled Trials (RCTs), Clinical Trials, Clinical Studies, and Observational Studies, with full text available and conducted on humans. Search strategies (strings adapted when necessary to fit the specific search requirements of each database) used the following Boolean expressions: keywords and terms: ((“sex”[tiab] OR “sexual”[tiab] OR “reproduct*”[tiab]) AND (“health”[tiab]) AND (“educ*”[tiab] OR “program*”[tiab] OR “intervent*”[tiab])) AND (“youth”[tiab] OR “adolescent*”[tiab] OR “teen*”[tiab]) AND (“school*” OR “communit*” OR “institution*”) AND (“survey” OR “questionnaire” OR “interview” OR “focus group*”) AND (“Satisfaction” OR “Perception” OR “Evaluations” OR “feedback”))(“2012/03/21”[Date-Entry]: “2022/03/21”[Date-Entry]). 

Moreover, we conducted a grey literature search of other papers, using hand searches of key conference proceedings, journals, professional organizations’ websites, and guideline clearing houses. Finally, with a snowball technique, we examined references cited in the primary papers to identify additional eligible papers.

### 2.2. Inclusion and Exclusion Criteria

The inclusion criteria were as follows: (1) language: articles written in English; (2) study design: Randomized Controlled Trials, Clinical Trials, Clinical Studies, and Observational Studies with original primary data; (3) population of interest: pre-adolescents and adolescents aged 11–19 living in the European Union; (4) intervention: sexual health interventions; (5) outcome measurement: perceptions and level of satisfaction with sexual health and reproductive health interventions in pre-adolescent and adolescent students; (6) comparison: not relevant, both studies with or without control groups were included. The exclusion criteria were: (1) articles not pertinent to the research topic; (2) populations of a different age or living outside the European Union; (3) studies with interventions, including prevention of unintended pregnancies only or prevention of STIs only; interventions including other topics also; (4) study protocol or other papers without original data. Table 1 summarized the PICOS (Patients, Interventions, Comparators, Outcomes, and Study Designs) eligibility criteria.

### 2.3. Data Extraction and Quality Assessment

Reviewers screened titles and abstracts and selected the eligible articles based on the inclusion and exclusion criteria. Full texts of all potentially eligible studies were retrieved after the removal of duplicates, then extracted and reviewed independently by the five reviewers (LD, AM, MM, CC, and YL) using a pre-tested data extraction form. Disagreements regarding the eligibility of the studies for inclusion were resolved by discussion among the researchers’ groups. The researchers, following standardized norms for literature collection, extracted data from the included studies. Details retrieved included: name of the first author, publication year, country, study design, population study with age, sex distribution and environment, type, time and duration of intervention, primary and other outcomes, and results stratifying the studies for the different outcomes. Results were reported as percentages for each satisfaction category or as mean ± SD where possible. Data extraction followed the methods provided by the Cochrane Reviewers’ Handbook [12]. 

Any disagreement was solved by consensus. The study authors or investigators were contacted when additional information was necessary [13]. Following PRISMA guidelines [11], we evaluated each included study for risk of bias. 

Selected studies were independently and blindly assessed for the risk of bias separately by three researchers (MM, AM, CC) using the recommended scale by Cochrane method bias. The “Version 2 of Cochrane risk-of-bias tool for randomized trials” was used to evaluate all the included randomized control studies [14], the “STROBE statement checklists for observational studies” was applied for the evaluation of cross-sectional or cohort studies [15], and the Risk Of Bias In Non-Randomized Studies of Interventions (ROBINS-I) [16] was used for quasi-experimental study. Any reviewer disagreements on the quality scores were resolved through discussion and, if necessary, a fourth blind reviewer (YL) was involved as a tiebreaker. The risk-of-bias evaluation was made on the basis of the primary outcome of interest: perceptions and level of satisfaction with sexual health interventions. This methodological choice was supported by the PRISMA guidelines [11].

The Cochrane risk-of-bias tool for randomized controlled trials analyzes five bias domains: (1) bias arising from the randomization process, (2) bias due to deviations from intended interventions, (3) bias due to missing outcome data, (4) bias in measurement of the outcome, (5) bias in selection of the reported result. The response options for the signaling questions in each domain were: yes/probably yes/probably no/no; no information. These categories provide the basis for an overall risk-of-bias judgment for the specific trial result being assessed in low risk of bias, some concerns, and high risk of bias. The STROBE statement is a 22-item tool divided into three different checklists: cohort study, cross-sectional and case report studies. In line with a previous study, we adopted a cut-off for three levels of score: 0–14 poor quality, 15–25 intermediate quality, and 26–33 good quality [15].

The ROBINS-I is a tool developed to assess the risk of bias in the results of non-randomized studies that compare health effects of two or more interventions. The ROBINS-I tool covers seven domains, providing a framework for considering any type of non-randomized studies’ effects of interventions. The first two domains address issues before the start of the interventions that are to be compared (“baseline”) and the third domain addresses classification of the interventions themselves. The other four domains address issues after the start of interventions regarding bias due to missing data; bias in measurement of outcomes; bias in selection of the reported result. The response options for each domain level were the same as RoB-2, but the overall assessment featured values of low, moderate, serious, critical, or no information on the risk of bias [16].

## 3. Results

### 3.1. Study Selection and Characteristics

**Figure 1:**
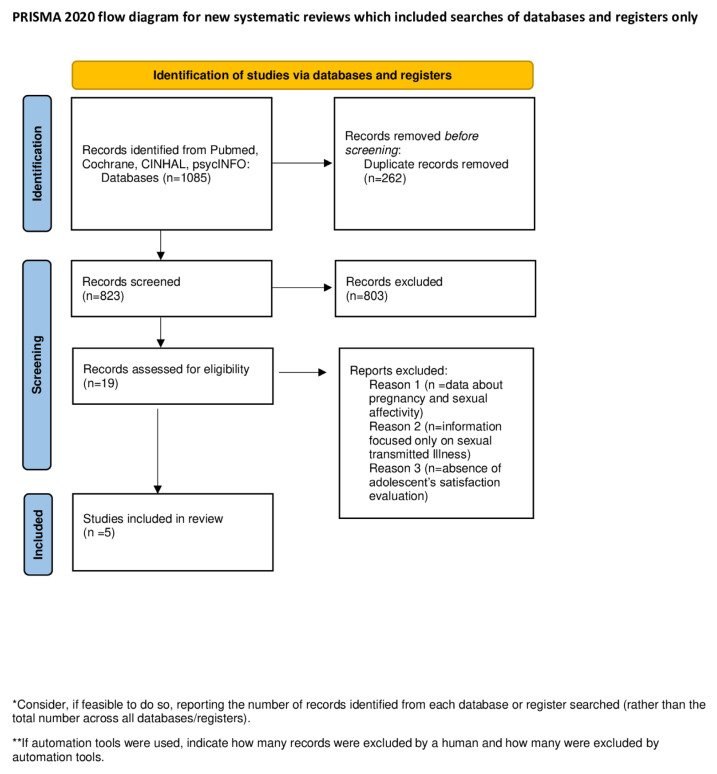
PRISMA Flow Diagram.

In total, 823 articles were extracted from the chosen database and followed our inclusion/exclusion criteria (Table 1). Studies from 2012 to 2022 were detected and, during the first step, we excluded 804 studies based on title and abstract reading. The main reasons for exclusion were linked to the studies with a non-UE setting and non-fitting study protocols. The second step consisted of full-text reading of 19 articles. Finally, only five papers were included in our review, as shown in Figure 1 (PRISMA flow diagram). Main reasons for exclusion after full-text screening were the availability of data inherent only in the prevention of unintended pregnancy, or only in affectivity, or only in information about STIs, in all cases, without covering an assessment of adolescent satisfaction. Among the included studies, study designs were heterogeneous: two papers were quasi-experimental [17,18], another two papers were RCTs [19,20], and one paper had an observational design [21].

The geographic origin of the articles was as follows: Italy (*n* = 2), Sweden (*n* = 1), Scotland (*n* = 1), and Finland (*n* = 1). The sample range varied from 168 to 902 participants, while ages ranged from 13 to 18 years. As to be expected from a study conducted in the school setting, the gender of the population was equally distributed; the only exception is in the work of Pakarinen and colleagues [21], where the male prevalence is 70.3%. The length of the SRH intervention varied from only one day to 4 months and the time of every single session from 45 min to 2 h. All included studies adopted questionnaires to evaluate final outcomes (e.g., student satisfaction).

### 3.2. Quality Assessment

We assessed the risk of bias of each study using different tools, as explained in the Methods section. Two of the included papers [17,18] had a quasi-experimental design and were assessed by the ROBINS-I scale, finding a moderate risk of bias. The ROB-2 scale was used to assess two RCT studies [19,20]. Finally, we included only one observational study [21] analyzed with the STROBE scale. In detail, a study by Benni et al. [17] was found to be of moderate risk, especially for bias in the measurement of outcomes, while the study of Mitchel et al. [18] was found to be generally of a low risk of bias, except for the bias due to missing data during analysis. Del Prete et al. was the only RCT study [19] that obtained a high risk of bias, mainly due to a lack of information in the randomization process. A study by Jelstrom et al. [20] was generally well conducted. However, it was assessed as “some concern” of risk because of the participants’ lack of blindness, although we are aware that when health promotion interventions such as SRH interventions are observed, it is almost impossible to make the participants blind.

Finally, the only observational study, by Pakarinen et al. [21], was rated as “intermediate level” of quality. Table 2 summarizes all quality assessment results.

### 3.3. Data Extraction

Study characteristics are presented in Table 3. The majority of the included studies considered participants’ knowledge and belief after the intervention as outcomes; however, following our systematic review aim, we assessed only results related to the perceptions of and satisfaction with SRH interventions. As previously mentioned, the interventions of the five included studies had very heterogeneous designs but were always evaluated through quantitative questionnaires and tools. In order to categorize the variability in the proposed interventions, we highlighted two intervention designs. The first kind of SRH education program was conducted by health or non-health research groups who administered the lessons and activities offered [19,20,21]. The second kind of SRH educational program was structured entirely following the peer-to-peer approach, with prior appropriate training and selection of coaches [17,18].

#### 3.3.1. Health- or Non-Health-Team-Led SRH Educational Programs

A study by Del Prete et al. was conducted in a secondary school. The intervention was planned by a team of health professionals with years of experience in counseling interventions targeting young people [19]. The intervention included three meetings, one and a half hours each, with students within the classrooms. In the first meeting, an explanation of the project and its aim was provided; then, entry questionnaires were distributed and collected, and the training began. The second meeting and the first part of the third followed a discussion of the subject areas covered by intervention: body awareness (e.g., physical and psychological aspects of puberty, anatomy, and physiology), relationship (e.g., friendship, love, affectivity, first sexual intercourse), counseling centers, contraception, and protection (e.g., contraceptive methods, sexually transmitted diseases). During the baseline assessment, 67.3% of respondents expressed an expectation to better understand sexuality through classroom meetings and with professionals, and it was found that expectations were met for 93.2% of the students who attended the meetings. Further, 28.8% of the intervention group suggested continuing these types of meetings.

The other RCT, proposed by Jelstrom et al. [20], tried to analyze the effect of an SRH program, implemented in high school, called SAFETY, and run by professional actors and staff from the municipality’s youth guidance center and school nurses. The SAFETY intervention also included games, exercises, and practical demonstrations.

The control group underwent standard education from school staff based on the sex education guidelines (e.g., human sexuality, reproduction, menstruation, love, sex, pregnancy). Students in the intervention group rated their experience and feelings about the program with seven four-level scale questions (from not at all accurate to very accurate). The majority (92%) of the students in the intervention group thought the play was good/very good. The play was considered entertaining (enjoyment 89%), and it was easy to get involved (identification) in the characters’ problems (80%). Students liked the fact that school staff took part in the program (82%).

Finally, considering studies with interventions led by professionals, we included a study by Pakarinen et al. [21]. The SRH intervention had three different components: theorical classroom lecture, informational materials (i.e., webpage, posters about safer sex, information leaflet about STIs and testing, HIV infection and testing, condoms), and condom distribution. Students were involved in a self-evaluation of the three parts of the intervention using a Likert scale from 1 to 5. Among the participants, 43.3% rated the lesson as satisfactory and about 42.5% as good or excellent. Participants also self-evaluated the classroom lesson in terms of learning and implementation. The average learning score from the classroom lesson was 3.4 ± 1.2, while implementation was 2.6 ± 1.0.

The self-evaluation of the classroom lesson was most frequently associated with the participant’s relational status. The majority of respondents (75.3%) read at least one of the sources of informational materials available during the intervention, especially the condom information pamphlet.

Condom distribution was rated positively by almost all the students. Participants reported that it was useful for condoms to be distributed in schools, and 40% felt that the distribution was well-organized. The mean score of the variable measuring the implementation of condom distribution was 2.3 ± 1.0.

#### 3.3.2. Peer-to-Peer Coaching-Conducted SRH Educational Programs

Regarding the peer-to-peer SRH educational program, we analyzed a study by Benni et al. [17] that investigated an SRH program performed by two quasi-peer coaches using interactive techniques (e.g., brainstorming, role playing, discussions). Normally, all peer coaches were trained before starting the teaching activities to ensure accuracy and similarity during intervention delivery. Specifically, in the study by Benni et al., coachers were 19 to 22-year-old volunteers and conducted 2 h sessions in each class focusing on anatomy and physiology of the reproductive system, STIs, contraceptive methods, voluntary interruption of pregnancy, and prevention.

Satisfaction questionnaires were completed by 99% of the students involved. Further, 87.06% of the respondents felt emotionally involved in the discussion during the lesson, 99.50% felt that the peer-to-peer approach was the right strategy to deal with sexuality issues, 90.89% felt wiser about sexuality, and 32.38% wanted to become peer coaches. The final phase of the intervention presented a discussion meeting with the school and the teachers. In general, teachers appreciated the intervention but suggested that it would be better to promote both sex and affective education. Teachers also reported the opinion of students’ parents, who generally showed good acceptance of the intervention. Only in one school did some parents dislike this educational strategy.

Finally, a study by Mitchel et al. [18] analyzed the effect of the STASH intervention conducted by peer students aged 14–16. In agreement with Benni et al.’s study, peer-to-peer coaches were trained before starting the SRH intervention. Moreover, this program included the creation of peer support groups on social media. Acceptability was monitored through a questionnaire. Thus, 74% of the exposed students stated that the way STASH was run was acceptable, while 78% said the information provided was acceptable.

## 4. Discussion

To the best of our knowledge, this is the first systematic review summarizing the available peer-reviewed evidence on perceptions of and satisfaction with sexual and reproductive health interventions in pre-adolescent and adolescent students in EU/EEA countries. This review describes the intervention designs used, the methods of evaluation, and the results obtained, along with their limitations and benefits, discussing them with the guidelines of leading international organizations in the field. Of the five studies included in the review, two had an RCT design, two were quasi-experimental, and one was an observational study. RCT and quasi-experimental designs are described as not sufficiently suitable for sexual education evaluation [22,23], and an overall intermediate quality among the included studies was blindly assessed by the research group using validated checklists. None of the articles included in the present review used qualitative methodologies, such as focus group or semi-structured interviews, to assess participant satisfaction. Furthermore, although experimental designs can provide estimates of satisfaction with SRH interventions, they provide limited insight into how and why the intervention was or was not appreciated by not focusing on different components or content in the intervention. As a result, the ability to compare the results of an included study with those of another setting may be compromised.

Although using process evaluation tools or reporting on the feasibility and acceptability of the SRH intervention provide valuable additions and a better understanding of the planning, implementation, and monitoring of the interventions [7], the lack of such studies is demonstrated by the results of the current review, in which only 5 studies out of 823 reviewed were included.

A variety of monitoring and evaluation tools have been developed in recent years and can be adapted to different contexts, such as the Sexuality Education Review and Assessment Tool [24] and IPPF’s Inside and Out [25]. However, although these tools are very useful for standardized evaluation of the content of SRH education programs and for examining and evaluating the comprehensiveness and quality of programs, they do not focus at all or only minimally on evaluating interventions in terms of participants’ perceptions and satisfaction.

All the articles included in this review showed excellent levels of satisfaction with SRH programs despite the variety of contexts (public and private schools), the age of the participants (13–18 years), the duration of the sessions (from 45 min to 2 h), the duration of the intervention (from only one session to 4 months), and the planning of the intervention (peer to peer, play, or lesson).

In a study by Del Prete et al., in which the interventions were proposed and conducted by healthcare personnel, the questionnaire administered at the end of the intervention revealed how expectations were met for 93.2% of students who took part in the meetings [19]. Much more importance was given to participants’ practical involvement, including games and exercises, in the program described in a study by Jelstrom et al., where the use of play as a tool to convey SRH education programs was appreciated by 92% of the participants. Even considering content, students involved in a more active way showed higher levels of knowledge, improved attitudes, and less risky behavior compared to the control group, which attended conventional SRH education lessons [20]. A study by Pakerinen et al. considered the evaluation of participants’ perceptions and satisfaction to be a fundamental tool for monitoring the quality of SRH education programs and for improving them. In addition, while still obtaining consistently good results, attention was paid to creating a questionnaire that would allow for evaluation of different phases of the program: classroom lessons, information materials, and condom distribution [21]. Benni et al.’s study also focused solely on assessing participants’ perceptions and satisfaction, yet it presented a uniqueness: it included moments of discussion by asking for feedback not only from participating students but also from teachers and parents. Positive results in adolescents’ satisfaction, highlighting the need for more youth-gathering places, were found. Working group members and educators generally provided positive evaluations, although difficult communication was perceived [17]. Finally, a study by Mitchel et al. investigated an innovative mode of an SRH program, involving social networks and the training of some peer supporters as influencers/trainers, again achieving high acceptance rates among both those who were trained and those who used the program [18].

In general, programs involving peer-to-peer approaches or hands-on activities seem to lead to higher levels of acceptance and satisfaction than conventional ones. Peer-to-peer education has become a popular strategy for health promotion, based on the assumption that young people learn and influence each other in both risky and safe behaviors [26,27,28,29].

In addition, SRH education programs administered early would also seem to increase levels of acceptance, as well as considering that this would meet the need to provide adequate SRH education prior to students’ first sexual intercourse. In fact, currently, most international institutions suggest promoting SRH education program starting from middle school.

Considering the high importance given by international organizations to the implementation of SRH educational programs in all the countries of the EU/EEA area [3], these results on students’ perception of and satisfaction with SRH education programs should be considered very encouraging for countries that still have a shortage of these interventions.

This systematic review has some limitations. Only studies published in English were considered, resulting in the exclusion of those published in other languages. In addition, this review did not include gray literature, such as UN reports and NGO-led studies, which often do not enter the peer-reviewed literature and, potentially, use different approaches. All the included studies were not assessed at low risk of bias and this aspect represents a limitation in the interpretation of the results.

## 5. Conclusions

This review explored the lack of studies that focused on participants’ evaluation of SRH education programs in terms of perception and satisfaction. Although RCTs and quasi-experimental designs are undoubtedly important for demonstrating the effectiveness of an intervention, qualitative study designs might be suitable for assessing the evaluation. A need for standardized tools that also include quality assessment and satisfaction with the implemented interventions remains. However, the limited data found in the scientific literature point to a generally high rate of participants’ satisfaction with SRH interventions, suggesting how these programs could be implemented and appreciated, even in EU/EEA countries that still have a shortage of them and in earlier school levels. A peer-to-peer and early SRH education program seems to achieve higher participant satisfaction.

## Figures and Tables

**Table 1:** PICOS inclusion and exclusion criteria.

Parameter	Inclusion Criteria	Exclusion Criteria
Population	Pre-adolescents and adolescents aged 11–19 living in the European Union.	Children aged 0–10, adults, workers.
Intervention	Sexual health and reproductive health interventions	Interventions including prevention of unintended pregnancies only or prevention of STIs only. Interventions including also other topics.
Comparator	None	None
Outcome	Perceptions of and level of satisfaction with sexual health interventions in pre-adolescent and adolescent students	Level of knowledge after sexual health intervention and incidence of STIs
Study design	Experimental, quasi-experimental or observational study with original primary data and full-text studies written in English	Study Protocol or other papers not presenting original data (e.g., reviews, letters to editors, trial registrations, proposals for protocols, editorials, book chapters, conference abstracts).

**Table 2:** Quality assessment of RCTs, quasi-experimental, and observational studies.

Authors	Study Design	Tool for Assessment	Quality
Benni et al. 2019 [17]	Quasi-experimental	ROBINS-1	Moderate
Mitchel et al. 2021 [18]	Quasi-experimental	ROBINS-1	Moderate
Del Prete et al. 2021 [19]	RCT	Cochrane ROB2 Tool	High
Jerlstrom et al. 2020 [20]	RCT	Cochrane ROB Tool	Some concern
Pakarinen et al. 2019 [21]	Observational	STROBE	(17.5/33) Intermediate

**Table 3:** Studies included in the review.

Author, Year, Country	Study Design	Study Population	Intervention	Outcomes	Results
Benni et al., 2015, Italy [17]	Quasi experimental study	N: 902 Intervention group (%): 547 (60.64) Control group (%): 355 (39.36) Age: 15.28 ± 0.61 Males (%): 399 (44.73) Setting: public and private schools	Type of intervention: Sexual health education course, performed by two near-peer educators in each class using interactive techniques. Duration and time: 2 sessions, 2 h per session	Primary outcome: Satisfaction evaluation Assessment method: anonymous satisfaction questionnaire consisting of eight closed-ended questions Other outcomes: Effectiveness evaluation (basal sexual knowledge, behaviours, beliefs and access to services for young people)	The 87.06% of responders felt emotionally involved in the discussion in the classroom, 99.50% pointed to peer education as the right way to deal with sexuality topics, 90.89% felt wiser in sexuality matters, 76.57% did not attend a group where they could continue to talk about sexual health and 32.38% desired to become peer educators
Jerlström et al., 2020, Sweden [20]	Randomized controlled trial	N: 826 Intervention group (%): 427 (51.69) Control group (%): 399 (48.31) Age: 15 Males (%): 409 (49.52) Setting: municipal schools	Type of intervention: SAFETY program led by professional actors divided in a play/theater, a value exercise, chlamydia games, condom school and a replay. A play-based activity, portraying youths and problems with condom use, information about chlamydia, STIs, emergency contraceptive pills and sexuality Duration and time: 1 session of 80 min	Primary outcome: Satisfaction evaluation Assessment method: web-bas survey with seven questions with four-step scale Other outcomes: knowledge of condom use and chlamydia	The 92% of responders felt the play was good/very good; 89% thought that was entertaining while 80% thought that was easy to get involved in the characters’ problems. Most students felt it was okay to change one’s mind even after having decided to do something. Students appreciated that school staff took part in the study
Mitchell et al., 2021, Scotland [18]	Quasi experimental study	N: 559 Peer supporter intervention group (%): 97 (17.35) Intervention group (%): 240 (42.94) Control group (%): 222 (39.71) Age: 14–16 Males (%): 231 (41.32) Setting: state-funded schools	Type of intervention: STASH Program, peer-led intervention focused on sexual health. Duration and time: 4 months	Primary outcome: Satisfaction evaluation Assessment method: web-based baseline, follow-up and control questionnaires; training evaluation, peer supporter questionnaire, semi-structured interviews, activity observations and monitoring log Other outcomes: self-efficacy and communication skills, increased autonomy and motivation, social support for healthy sexual behaviour	The 74% of exposed students said the way STASH was run was acceptable, 78% said the information provided was acceptable.
Del Prete et al., 2012, Italy [19]	Randomized controlled trial	N: 322 Intervention group (%): 147 (45.65) Control group (%): 175 (54.35) Age: 13–14 Males (%): 162 (50.30) Setting: municipal secondary schools	Type of intervention: Sexual health education course based on counseling interventions, Duration and time: 3 sessions, 1.5 h per session	Primary outcome: Satisfaction of intervention and communication with family, social group, and health professionals on the topic of affectivity Evaluation of the family perception. Assessment method: Questionnaire about satisfaction and perception after the intervention Other outcomes: Personal knowledge about sexual transmitted diseases (STDs) and methods of prevention and contraception, contraception, sexuality, affectivity along with the perceived level of knowledge on the topic.	Prior to the intervention, 67.3% of respondents expressed an expectation to better understand sexuality through classroom meetings, and the survey showed that these expectations were met for 93.2% of students who participated in the meetings. 28.8% of the intervention group suggested continuing these types of meetings, and 28.1% of them have no suggestions for practitioners.
Pakarinen et al., 2019, Finland [21]	Observational Study	N: 168 Age: 16–18 - Intervention group (%): 169 (100%) - Control group (%): Missing Males (%): 111 (70.30 *) Setting: state-funded schools vocational institutions	Type of intervention: Classroom lessons with information about sexuality, sex, safer sex, condom use and STIs. The lessons was based on the sexual education materials for adolescents produced by Hivpoint (former Finnish AIDS Council). The intervention was also composed by free condom distribution and informative materials: web page, a poster about safer sex; an information leaflet about condoms, STIs, HIV infection and their testing; Duration and time: 11 weeks, 45min per session	Primary outcome: Satisfaction evaluation Assessment method: self-completed electronic questionnaire on quality of (a) classroom lessons, (b) information materials and (c) condom distribution. Other outcomes: characteristics associated with the self-evaluation of the sexual health promotion intervention. Possible implementation in school environment	(a) Self-evaluation of the classroom lessons: - Satisfaction score: 3.35 Learning score: 3.4 (SD: 1.2), - Implementation score: 2.6 (SD: 1.0) (b) Self-evaluation of information materials Learning score: 3.3 (SD: 1.2) (c) Self-evaluation of condom distribution Implementation score: 2.3 (SD: 1).

* percentage was calculated on 155 participants due to missing data.

## Data Availability

Not applicable.
